# UK newspapers ‘on the warpath’: media analysis of general practice remote consulting in 2021

**DOI:** 10.3399/BJGP.2022.0258

**Published:** 2022-10-04

**Authors:** Gilly Mroz, Chrysanthi Papoutsi, Trisha Greenhalgh

**Affiliations:** Nuffield Department of Primary Care Health Sciences, University of Oxford, Oxford.; Nuffield Department of Primary Care Health Sciences, University of Oxford, Oxford.; Nuffield Department of Primary Care Health Sciences, University of Oxford, Oxford.

**Keywords:** general practice, media analysis, printed media, remote consulting, workload

## Abstract

**Background:**

Following a large-scale, pandemic-driven shift to remote consulting in UK general practice in 2020, 2021 saw a partial return to in-person consultations. This occurred in the context of extreme workload pressures because of backlogs, staff shortages, and task shifting.

**Aim:**

To study media depictions of remote consultations in UK general practice at a time of system stress.

**Design and setting:**

Thematic analysis of national newspaper articles about remote GP consultations from two time periods: 13–26 May 2021, following an NHS England letter, and 14–27 October 2021, following a government-backed directive, both stipulating a return to in-person consulting.

**Method:**

Articles were identified through, and retrieved from, LexisNexis. A coding system of themes and narrative devices was developed iteratively to inform data analysis.

**Results:**

In total, 25 articles reported on the letter and 75 on the directive. Newspaper coverage of remote consulting was strikingly negative. The right-leaning press in particular praised the return to in-person consultations, depicting remote care as creating access barriers and compromising safety. Two newspapers led national campaigns pressuring the government to require GPs to offer in-person consultations. GPs were quoted as reluctant to return to an ‘in-person by default’ service (as it would further pressurise a system already close to breaking point).

**Conclusion:**

Remote consultations have become associated in the media with poor practice. Some newspapers were actively leading the ‘war’ on general practice rather than merely reporting on it. Proactive dialogue between practitioners and the media might help minimise polarisation and improve perceptions around general practice.

## INTRODUCTION

UK general practice is under enormous strain, driven by unsustainable workloads, recruitment, and retention difficulties, especially in deprived areas, and a significant COVID-19 backlog.^1^^,^^2^ At the time of writing, an inquiry by the parliamentary Health and Social Care Committee is ongoing to identify underlying challenges and ways forward for the future of general practice.^3^ This includes an assessment of the potential and limitations of remote consulting (for example, online forms, email, phone, or video), as this complex process of service reorganisation led to practical and ethical dilemmas during the pandemic, including around access and efficiency.^4^

Policy on GP consultations in the UK has undergone several shifts during COVID-19. The initial move from in-person to remote consulting was first introduced in March 2020 as part of infection control measures.^5^ Almost overnight, GP surgeries began offering clinical assessments remotely, with in-person appointments offered only in specific circumstances, in what was widely considered a temporary response to the crisis. Yet in July 2020 the government announced that a remote-first approach was to become default policy in England,^6^ at a time when the first wave of the pandemic had receded and many restrictions had been lifted. This was met with surprise and criticism by the media, as the authors of the current study have demonstrated in previous studies.^7^^,^^8^

As the pandemic progressed and pressure on the health service intensified, in May 2021 NHS England encouraged a return to in-person care in general practice whenever safe to do so.^9^ Later that year the new Secretary of State for Health and Social Care strongly supported this move and urged GPs to offer more in-person appointments,^10^ a sentiment echoed by the Prime Minister.^11^ In the context of ongoing condemnation of GP remote consulting in the media, the government released a directive, including a £250 million investment, to improve capacity for in-person consultations in general practice (see Box 1 for more details on policy changes).^9^^,^^12^

**Box 1.** Policy changes

**NHS England — letter** On 13 May 2021 — when the second wave of the pandemic in the UK (largely driven by the Alpha strain of SARS-CoV-2) had subsided — NHS England announced that GP surgeries had to ensure they were offering in-person appointments, with patients being allowed to choose whether they see a GP in person or remotely.^9^ In-person care could, however, be denied where there were *‘good clinical reasons to the contrary’*,^9^ such as the presence of COVID-19 symptoms. GP reception areas would once more be open to the public to enable those without easy access to phones or other devices to book an appointment, but physical distancing would remain in place. These instructions contradicted guidance from Public Health England, also published on 13 May 2021, reminding the public to *‘work from home where you can’*.^14^ **NHS England — government directive** On 14 October 2021, NHS England, in collaboration with the Department of Health and Social Care, released its directive to improve GP access, with focus on increasing the number of in-person consultations.^9^^,^^12^^,^^15^ The directive followed ‘Freedom day’ on 19 July 2021 when, despite the high levels of Delta strain circulating in the community, most restrictions (for example, limits on venue capacity, physical distancing, and mask wearing in most situations) had been lifted and the public was encouraged to discontinue remote working.^2^^,^^16^ More comprehensive than the May letter, the main component of the directive was a £250 million winter access fund available to GP surgeries that submitted applications stating how they would endeavour to increase the number of in-person consultations during the upcoming months. The funding could be used to improve access by, for example, hiring more locum GPs, other healthcare professionals (for example, nurses, physiotherapists, and podiatrists), and/or administrative staff. Other aspects of the plan included short-term solutions, such as reducing GPs’ administrative tasks and enabling pharmacists to prescribe medication for minor ailments, and long-term solutions, such as recruitment and retention initiatives for GPs and other primary care health professionals. Other components included ‘levelling up’ GP services through measures such as patients rating their experiences, and the public release of performance data; improved communication to help patients understand how to access primary care for their specific needs; and a zero-tolerance campaign on violence and abuse towards NHS staff.

Comments by the media and government ministers suggesting that GPs were offering fewer appointments (and even sometimes conveying the impression that surgeries were ‘closed’) did not reflect what was happening in practice. Although the number of in-person appointments at the time of this current study in 2021 was lower than pre-pandemic levels (5.3 million in August 2021 versus 8.8 million in February 2020), the number of telephone consultations almost trebled from 2.2 million in February 2020 to 6.2 million in August 2021.^13^ As such, the total number of consultations increased from 11 million to 11.5 million, albeit with a higher proportion undertaken remotely.

Building on the research the authors had conducted into media representations of remote consulting during the first year of the pandemic (in 2020),^7^^,^^8^ this current study examines newspaper coverage in 2021, and how this has evolved alongside policy (a return to in-person consulting) and wider media discourse (a rise in anti-GP rhetoric).

**Table:** How this fits in

In 2020, the shift from in-person to remote consulting in general practice was depicted positively by the media as part of the ‘war’ on COVID-19. In 2021, remote consulting was depicted negatively by the media, and linked in press articles to difficulties accessing primary care and compromises in patient safety. Newspapers led campaigns that successfully put pressure on government to require a return toin-person consultations.

In this study the following research questions are addressed.

- How did national UK newspapers cover the policy shifts away from the initial remote-first approach towards a return to in-person GP consulting in 2021?
- How were messages around remote and in-person consulting framed?
- What metaphors, tropes, and other narrative techniques were used, and what do these symbolise?
- What are the differences in how the various newspapers reported on GP consultations?

## METHOD

This study followed a qualitative approach to thematic analysis, which has successfully been used in previous research on news media representations of general practice.^8^^,^^17^ The research team consisted of one postdoctoral researcher in languages, one social science academic, and one GP academic. From the authors’ previous research and general reading, the authors were aware that the media had covered remote consultations fairly positively in the past but had negatively portrayed general practice. Although the authors inevitably brought these perspectives and knowledge to bear on the research, the design of the study (a systematic sample, formally analysed, and with extensive team deliberation) helped minimise researcher biases.

Nine newspapers were searched in this study: eight of the most widely read national newspapers in the UK — *Daily Mail*, *The Guardian*, *Express*, *The Times*, *The Telegraph*, *The Sun*, and *Mirror*, as well as their Sunday editions — and *The Voice*, self-described as ‘Britain’s favourite Black newspaper’. Together they comprise a wide range of political opinions and are read by different socioeconomic groups. They were the same newspapers analysed in the authors’ previous study,^8^ with consistency facilitating comparative analysis. Articles were collected from LexisNexis (https://www.lexisnexis.com/uk/legal/news) (for the eight national newspapers) and *The Voice*’s website (https://www.voice-online.co.uk).

The search terms used were ‘GP(s)’ combined with eight further terms: ‘video’, ‘remote’, ‘virtual’, ‘digital’, ‘online’, ‘phone’, ‘telephone’, or ‘face-to-face’. These are the same search terms as used in the authors’ earlier study, with the exception of ‘face-to-face’, which is a new addition based on the shift in language.

Articles were retrieved for two time periods in 2021, each beginning with a policy change news peg: the first (13–26 May) with NHS England’s letter,^9^ and the second (14–27 October) with the NHS England government directive^12^ (Box 1). Although debates around GP consultations have featured in the media throughout 2021, these two news pegs were chosen to consider newspaper coverage of the specific policy announcements. The duration of both time periods is 2 weeks, as previous research on this topic shows that media interest in a news peg tends to diminish after the first week.^8^ A third period, beginning with another letter from NHS England, dated 13 December 2021, regarding preparing the NHS for an anticipated increase in consultations as a result of the new Omicron variant of COVID-19 and other winter pressures,^18^ was also considered. However, as consultation format did not feature in the letter, this period was not included in the current study.

**Box 2.** Example quotes

**The push for a return to in-person consultations** ***1A: Negative experience: access*** *‘I’m lucky I have a smart telephone and I just press redial. But a lot of elderly people don’t have that. If there’s no one to fight their corner, they will just go under the radar.’* (Daughter of patient; *Express*, 15 October) ***1B: Negative experience: safety*** *‘He should never have gone to A&E in that condition. It is something that should have been sorted out way before then and, having approached his GP practice on four occasions, not to see him I think is the primary reason that they failed to recognise his condition and treat it* […] *I wish David had had Covid. If he had had Covid, he would have been treated. That’s the irony. How do you diagnose an ear infection without looking in the ear?’* (Father of patient; *The Times*, 18 October) ***1C: Positive experiences*** *‘For a significant proportion of patients, the blended model has been welcomed: the mother who sends in photos of her boy’s eczema so he doesn’t need to miss school; the teenager with crippling social anxiety who cries quietly down the phone; the elderly lady with a urine infection who does not want to sit in a waiting room that has become a petri-dish for bugs. We do not hear of these stories in the media.’* (Nishma Manek, GP; *The Guardian*, 14 October) **Policy changes and escalating conflict** ***2A: The media’s contribution to the policy changes*** *‘With breathtaking* [sic] *cynicism, Boris Johnson and Sajid Javid have taken a campaign by the Tory-supporting Daily Mail and Mail on Sunday and turned it into government policy. For months, the newspapers have been demanding that GPs return to seeing more patients face to face. Currently, just under 60% of appointments are in person, compared with 80% before the pandemic, with the rest taking placeon the phone or online. The newspapers decided that this isn’t what the public wants. This week, Mr Javid retreated into his Whitehall office to take aim at medics.’* (Journalist; *The Guardian*, 14 October) ***2B: GP response to May letter*** *‘Every practice will have different issues, with different levels of capacity and demand. But this letter does not include details of any extra investment or resource for primary care services, and does not acknowledge how exhausted and overstretched the whole workforce is.’* (Graham Jackson, GP and senior clinical adviser at the NHS Confederation; *The Times*, 15 May) ***2C: GP response to October directive*** *‘After weeks of promising an “emergency package” to rescue general practice, we’re hugely dismayed that while additional funding has been promised the package as a whole offers very little and shows a government completely out of touch with the scale of the crisis on the ground* […] *GPs and their teams will now be facing the worst winter for decades, and as a result, patients’ care will suffer. Appointments willbe harder to book, waiting times will get longer, more of the profession could leave and GPs will struggle to cope.’* (Richard Vautrey, chair of the British Medical Association’s [BMA’s] GP committee, in BMA press release; *The Guardian*, 14 October) **(Mis)representation and abuse of GPs** ***3A: Negative representations*** *‘GPs are on £100 000 plus a year and, yes, they’re busy. But if they didn’t realise people get sick all the time and that they’d be busy — they shouldn’t have gone into medicine. Sajid Javid is right to call them out on this. And doctors have no right to be affronted at being told to do what’s best for their patients. I know there’s a GP shortage so why then are so many of them choosing to go part time? Why make a bad situation worse?’*(Journalist; *Express*, 16 October) ***3B: Positive representations (and response to negative representations)****‘GPs and our teams have worked our socks off throughout the pandemic, delivering essential care and services when many other parts of the NHS shut* […] *Yet over the last couple of months, GPs and our teams have been subjected to a torrent of unfair and frequently offensive criticism from certain parts of the UK media and some politicians. It has been the worst I can remember in 30 years as a GP.’* (Martin Marshall, Royal College of General Practitioners chair; *Mirror*, 14 October) ***3C: Abuse****‘Abuse has become endemic. Last month, four practice staff in Manchester were hospitalised — one with a skull fracture — from a disgruntled patient. I’ve been screamed and shouted at down the phone on more occasions than I can count. Much of this abuse has been legitimised by some sections of the media with their clickbait anti-GP campaign. Politicians have not been blameless.’* (Ellen Welch, GP and author; *The Guardian*, 14 October)

In contrast to the early stages of the pandemic, a large volume of articles focused on or substantially discussed remote GP consultations. For this reason, articles that referred only briefly to the topic were excluded from the sample. Included articles could be news or opinion pieces, but blogs and letters to the editor were excluded.

Following this data collection, thematic content analysis was used to categorise articles according to themes or topics.^19^ The lead author read all the articles collected, identifying key themes and narratives, which were then brought together under five main categories. These were chosen both deductively, based on those found in the authors’ previous work including empirical research on remote consulting,^4^^,^^8^ and inductively, based on the present data.

A second researcher reviewed all articles and their categorisation. Both authors discussed their analyses to guide thematic synthesis and ensure consistency. The five categories were then revised and, in places, amalgamated, to present a comprehensive picture of the media’s coverage of GP consultations under three main storylines.

## RESULTS

### Description of the data

Period 1 contained 25 articles that either focused on or substantially discussed remote versus in-person GP consultations, limited to four newspapers: *The Telegraph*, *The Times*, *Daily Mail*, and *Express*. Of these, 19 were written in response to NHS England’s letter, whereas the remaining six did not acknowledge it. Period 2 contained 75 articles, all in response to the NHS England government directive. Articles appeared in all newspapers, with the exception of *The Voice*, which did not publish any articles on GP consultations during the timeframes chosen for this study (Table 1).

**Table 1.** Distribution of articles from both time periods across the newspapers

**Publication**	**Period 1, 13–26 May 2021**	**Period 2, 14–27 October 2021**
*Daily Mail*	1	19
*Express*	7	8
*The Guardian*	0	13
*The Independent*	0	6
*Mirror*	0	6
*The Sun*	0	3
*The Telegraph*	11	11
*The Times*	6	9
*The Voice*	0	0
Total	25	75

### Findings from the thematic analysis

Qualitative analysis led to the identification of three key storylines in the media’s coverage of GP consulting and relevant policy changes:

- the push for a return to in-person consultations;
- policy changes and escalating conflict; and
- (mis)representation and abuse of GPs.

#### The push for a return to in-person consultations

Many articles across both time periods drew attention to a need for better access to GP care and more in-person consultations. Various metaphors were used to emphasise depictions of in-person consultations as scarce in relation to demand. One journalist depicted GP appointments as being *‘like gold dust and face-to-face appointments even rarer’* (*The Times*, 24 October), another described in-person consultations as being *‘as rare as hen’s teeth’* (*Daily Mail*, 16 October), and a third suggested that *‘securing a face-to-face consultation feels like hitting the jackpot’* (*Daily Mail*, 14 October).

Two main arguments, illustrated by patient stories, were emphasised to justify the need for a return to in-person consulting. The first concerned the potential for widening health inequalities, primarily attributed to access disparities with remote-first GP care, particularly for patients who were older, had disabilities, or were homeless. According to one article, *‘vulnerable patients are struggling to get help, with some phoning more than 100 times a day to secure an appointment’* (*The Telegraph*, 13 May). This argument was supported by the story, widely circulated, of a daughter who called a GP surgery 286 times to book her 84-year-old mother an urgent eye appointment, only to be offered a phone consultation (Box 2, quote 1A).

The second argument concerned risk and safety in remote care, with articles focusing their narratives on missed symptoms and misdiagnosis. Concerns reported by the Royal College of General Practitioners (RCGP) that remote-first care *‘risks serious illnesses being missed’* (*The Times*, 13 May) were echoed by many journalists. Some articles took this stance further by suggesting that remote consulting may *‘have contributed to the death of some patients’* (*The Independent*, 14 October). To support this suggestion, two stories containing fatal outcomes appeared frequently. The story of the death of a 26-year-old from meningitis appeared in seven of the eight newspapers included in the current research. The articles reported that, during four remote consultations with GPs and nurses, in which the patient presented *‘with a range of escalating symptoms’* (*The Independent*, 18 October), none picked up his mastoiditis, which went on to cause a brain abscess. His father questioned the system (Box 2, quote 1B). The story of a 69-year-old also featured widely. Having been ‘refused’ in-person consultations, she was allegedly informed in a phone consultation that her leg and hip pain was arthritis; by the time she was diagnosed with cancer *‘it was too late for life-saving surgery’*. The article argued that *‘cancer is often diagnosed after a GP has noticed subtle symptoms* […] *extremely difficult to pick up on a Zoom call’*. It offered a fatalistic stance on remote care: *‘it wasn’t the* [corona] *virus that killed the former PE teacher three weeks ago, but the growing use of “telemedicine”’* (*Express*, 14 May).

No articles from either time period reported on positive patient experiences. Only one article offered a more nuanced view on when remote consultations may be appropriate and reminded the reader that they do happen for a wide range of people and conditions; however, as these conditions tend to be minor and the outcomes positive, *‘*[we] *do not hear of these stories in the media’* (*The Guardian*, 14 October) (Box 2, quote 1C). When articles pointed to the benefits of remote consulting in general practice, these tended to be limited to them being *‘a lot less of a faff than going to the surgery in person’* (*The Telegraph*, 15 October).

The media’s mobilisation of patient stories to reinforce their push for a return to in-person GP care seemed to be underpinned by a deterministic logic. At the start of the pandemic, the shift to remote care was widely considered a *‘perfectly sensible’* measure (*Daily Mail*, 14 October) *‘to protect patients and staff’* (*The Times*, 18 May) — the remote modality was thus depicted to a significant extent as causing safer care. Remote consulting — whether by phone, video, and/or email — was described as having the potential to save lives, but the wider context in which this could be realised (such as the significant work required by healthcare staff to enable a safe standard of remote care) was often overlooked by the press.^8^

An equally deterministic logic was adopted in 2021, but remote consulting was now depicted as causing safety incidents, and in-person appointments described as being the ones that *‘save lives’* (*The Independent*, 14 October), whereas other factors, such as long waiting lists in secondary care, and the need for additional tests and appointments to reach a diagnosis, were neglected. As the stories of patient experiences show, these deterministic narratives on remote consulting have also become fatalistic, with descriptions of the *‘harrowing consequences of diagnosis by phone, email or screen: symptoms missed, treatments delayed, lives ruined — and, too often, tragically lost’* (*Daily Mail*, 15 October).

#### Policy changes and escalating conflict

Negative patient stories were often mobilised in the context of extensive campaigns, particularly by *The Telegraph* and *Daily Mail*, calling for more in-person consultations — so vehemently that they led *The Guardian* to describe the newspapers as *‘on the warpath’* against remote consulting (*The Guardian*, 15 October). These campaigns played a key role in influencing the government to effect policy changes, which were widely praised by large portions of the media (Box 2, quote 2A).

When the May letter was published, only the right-leaning media (*The Telegraph*, *The Times*, *Express*, and *Daily Mail*) saw it as newsworthy and reported on it in the sample included in the current study. In addition to welcoming the policy change, *The Telegraph* used the opportunity to congratulate itself and its readers for their contribution towards it: *‘Congratulations, everyone! Well done us’* (*The Telegraph*, 19 May) (Box 3).

**Box 3.** The media’s influence on policy

***The Telegraph*, 19 May 2021** *‘Congratulations, everyone!* *Well done us. All it took for NHS England to alter its guidance and tell GPs that they should offer patients face-to-face appointments was an immensely moving account from* [reader] *about the preventable death of his darling wife* [name] *, a couple of mortars fired by your columnist, Gunner Pearson, and several thousand emails of frustration, pain and disbelief from our magnificent Telegraph family. Plus, of course, a front-page scoop by Laura Donnelly and Rosa Silverman, which confirmed what we all suspected: doctors had been told to discourage patient appointments in person to promote digital consultations.* *The swift U-turn was both an important victory for common sense — not seeing patients may well be “dangerous”, as so many medics pointed out — and proof that enough people acting together can push back against alienating measures which rob our society of the personal touch we treasure.’* ***Daily Mail***, **14 October 2021** *‘LAST month, the Mail launched an ambitious and important crusade.* *Concerned so many readers and their loved ones were enduring the depressing ordeal of trying — and failing — to visit their family doctor, we delivered a powerful message to ministers: Let’s See GPs Face To Face.* *Today, four weeks on, this paper is proud to record another stunning triumph. For Sajid Javid is unveiling sweeping healthcare reforms which should make it easier to get an appointment in person. In these pages, the Health Secretary and Prime Minister pay tribute to our important campaign highlighting this scandal.’*

The October directive was also praised in the right-leaning press. Like *The Telegraph*, *Daily Mail* congratulated itself for its contribution, describing the directive as *‘a major victory for the Daily Mail’s Let’s See GPs Face to Face campaign’* (*Daily Mail*, 14 October) (Box 3). The newspaper even *‘welcome*[d] *the threat to penalise surgeries financially for conducting too many digital consultations’* (*Daily Mail*, 14 October). In contrast, the left-leaning press was critical of the move, echoing GP concerns (below) around pressures facing general practice. One article went further, suggesting that it was *‘irresponsible’* for *‘Mr Javid to encourage the public to* […] *insist on their right to a particular service’*, before describing the £250 million winter access fund a *‘slap in the face’* as insufficient to cover resourcing needs (*The Guardian*, 14 October).

The return to in-person consulting also generated conflict between GPs and policymakers. According to newspaper coverage, GP responses to the May 2021 letter were largely negative. It was reported that, although GPs welcomed the move away from an indefinite remote-first policy, they valued autonomy in deciding what was feasible and within capacity for optimal patient care. One of the articles described GPs as *‘angered’* by the letter and at NHS bosses for *‘adding fuel to falsehoods’* that surgeries were not offering in-person appointments (*The Times*, 15 May). Martin Marshall, RCGP chair, was quoted in several articles saying that *‘this is good news and is what patients and GPs want to see’* (*Express*, 15 May), but his support was more for *‘shared decision-making between GP and patient’* rather than a blanket return to in-person consultations (*The Telegraph*, 14 May). Only one article quoted the RCGP as describing the letter as *‘misjudged’* and *‘demoralising’* (*Express*, 20 May). Richard Vautrey, British Medical Association (BMA) GP committee chair, was quoted describing the letter as *‘completely tone deaf’*, with the BMA reportedly calling on NHS bosses to *‘clarify’* how GPs were to meet patient demand (*The Times*, 15 May) (see Box 2, quote 2B).

GP responses to the October directive were reported in the media as almost exclusively negative. In addition to general criticism of the directive as a whole, such as Richard Vautrey’s description of the plan as a *‘shambles’* (*Mirror*, 20 October) from *‘a government completely out of touch with the scale of the crisis’* (*The Guardian*, 14 October) (Box 2, quote 2C), more specific criticism targeted the £250 million winter access fund. Although some GPs — like Graham Jackson, then co-chair of NHS Clinical Commissioners — described the investment as *‘a welcome starting point’*, the consensus was that it would not *‘solve some of the longer-term workforce challenges’* (*The Times*, 14 October). They emphasised how the shortage of family doctors in the UK meant funding would not necessarily lead to recruitment as, according to one Shropshire GP, *‘there is no magic locum tree’* (*Daily Mail*, 15 October). They drew attention to more GPs retiring early or going part-time because of high stress levels and burnout, as well as fewer junior doctors training to become GPs (because of negative media coverage of the profession, see below) as key drivers of the GP shortage. The policy plan for league tables to ‘name and shame’ GP surgeries offering the fewest in-person consultations was also widely criticised. Although intended to increase the number of in-person appointments, GPs feared that the measures instead risked doing the opposite by adding to workloads and stress levels.

#### (Mis)representation and abuse of GPs

In the articles analysed there was mention of GPs traditionally viewed as *‘decent, dedicated and hard-working’* individuals (*Daily Mail*, 23 October) who *‘take great pride in making their communities healthier and happier’* (*Daily Mail*, 15 October). However, these views tended to be voiced primarily by members of the healthcare profession, such as by Chris Whitty, the government’s chief medical officer for England, who, speaking at the RCGP’s annual conference, described the work of GPs during the pandemic as *‘outstanding’*, adding that he was *‘massively admiring of what you all have done, and continue to do, in the biggest public health challenge in our professional career’* (*The Guardian*, 14 October). Some journalists also praised GPs for their service during the pandemic, with descriptive terms including *‘outstanding’*, *‘heroic’*, or *‘marvellous’* (*Daily Mail*, 16 May; 23, 27 October). Notably, these positive representations were almost exclusively limited to individual doctors who invited their patients, reportedly without hesitation, for an in-person appointment.

More frequently, GPs as a collective were berated in the media. Negative narratives around GP access — particularly the difficulty in securing an in-person appointment, with many patients being offered a remote consultation instead — led to the perception that GPs are *‘lazy’* (*The Guardian*, 15 October), reluctant to work, and/or care little for their patients: *‘it just seems like GPs want to do as little as they can’* (*The Telegraph*, 14 October). These perceptions led one journalist to encourage GPs to *‘start doing what they’re paid to do — SEE patients’* (*Express*, 23 October). In the context of policy changes to encourage patient feedback (Box 1), one article commented on how patients would be asked to rate their appointment experience via text message, like they would *‘an Amazon or pizza delivery driver’* (*Express*, 17 October).

Whereas most of the criticism was in response to the use of remote consultations, some articles used the policy announcements as a platform to criticise GPs more generally. A common perception was that GPs are *‘a bunch of overpaid part-timers’* (*Express*, 16 October). The same article criticised GPs for struggling with their high workloads and blamed them for exacerbating the GP crisis: *‘I know there’s a GP shortage so why then are so many of them choosing to go part time? Why make a bad situation worse?’* (Box 2, quote 3A).

Ironically, one of the reasons GPs are choosing to go part-time or leave the profession entirely is what Marshall has described as *‘the widespread vilification of hard working GPs’* by *‘some politicians and sections of the media’* (*The Independent*, 14 October) (Box 2, quote 3B). By criticising portions of the media for their vehement, and unfounded, criticism of GPs, such comments serve to offset, or even reverse, their negative representations of family doctors. They also segue into concerns around the negative effects of the media’s ‘demoralising’ comments, which, according to Marshall, not only have *‘an impact on the mental health of GPs’*, but have also led to *‘practice staff being on the receiving end of abuse’*, both verbally and physically (*Mirror*, 14 October) (see Box 2, quote 3C). Some family doctors warned that the introduction of GP league tables would not only increase workloads and stress levels (as noted above), but also lead to an escalation of abuse.

The government, aware of this abuse, responded by including a *‘zero-tolerance’* campaign to tackle abuse and harassment of GPs in their plan (*The Telegraph*, 18 October). However, one GP suggested that *‘any further demonisation of general practice will perpetuate the problem we are trying to address’* (*The Guardian*, 14 October). As such, an end to the government and media’s denigration of general practice may negate the need for such a campaign in the first place — while also, perhaps, beginning to alleviate the ‘GP crisis’ by improving both morale and GP–patient relationships.

## DISCUSSION

### Summary

This study has examined UK newspaper coverage on remote consulting in 2021, and how this evolved alongside a policy drive for increasing the number of in-person consultations in general practice. Media reporting featured concerns around inequity of access (for example, difficulties in securing an appointment) and patient safety (for example, the potential for missed symptoms and misdiagnosis) in remote care, illustrated through patient stories where detrimental outcomes were attributed to consultation format. Motivated by these concerns, portions of the right-leaning press led extensive campaigns for improved access in general practice, and subsequently praised the policy shift for in-person consultations. In contrast, GPs and GP representatives (and some journalists in the left-leaning press) were quoted as criticising policy changes for neglecting general practice pressures, including unmanageable workloads, high stress levels, and resource and staffing issues. Throughout 2021 remote consulting became increasingly contentious, creating tensions between government ministers, the media, GPs, and patients. A significant rise in anti-GP rhetoric was observed in the current study, with some journalists using remote consulting as a platform to criticise GPs for not fulfilling their professional duties, and thus further fuelling the GP crisis.

The absence of positive stories about remote consultations in the current sample may be partly explained by the fact that several newspapers were not merely reporting on remote consultations but campaigning to reduce them, and any positive stories would diminish that argument. But it may also be because of their perceived lack of newsworthiness. According to William Labov, narratives concerning *‘matters of life and death* […] *are identified with a high degree of reportability’*; positive patient experiences would therefore be considered less ‘reportable’. Labov adds that *‘credibility is inversely related to reportability’* [emphasis in original] — in other words, there is an inherent reporting bias against experiences where nothing went wrong.^20^

Whereas remote consultations were depicted positively (on one occasion as ‘miracles’) in the early weeks of the pandemic,^7^^,^^8^ in this period technology was cast in the role of ‘hero’, which — at least partially — saved society from a deadly pandemic. A year later, technology had become something of a villain, distancing the patient from the doctor’s therapeutic touch. These simplistic, polarised narratives around modes of consulting were not helped by a commodification of clinical care (patients were depicted as customers who had a right to see the GP in person). The reality of clinical diagnosis and management is, of course, far more nuanced, with multiple interacting influences contributing to safe and effective clinical care and the balancing of good care and patient satisfaction.^4^^,^^21^^,^^22^

### Strengths and limitations

The only previous explorations of this topic, to the authors’ knowledge, are the analyses of newspaper coverage from 2020;^7^^,^^8^ this current study offers an updated analysis of newspaper articles published in 2021. A wide range of search terms was used to collect 100 articles with strong focus on remote and/or in-person GP consultations from an extensive database (LexisNexis). The large number of articles, from eight widely read national newspapers with different political leanings, and from two time periods, facilitated broad analysis by enabling the authors to consider differences in media coverage, both collectively and by newspaper, in response to specific policy changes and over time.

Despite the variety of search terms used and the extensiveness of LexisNexis, there is a risk that some articles were missed. For scoping purposes, only articles from national UK newspapers from two 2-week periods in 2021 were used (a third period, at the start of the UK’s Omicron outbreak, was also considered, but rejected because of a lack of articles relevant to GP consultation format).

### Comparison with existing literature

Previous research on media representations suggests that public healthcare systems are often depicted in a negative light, with dramatic stories about inadequacies and inefficiencies outweighing positive ones.^23^ Drawing on Bourdieu, Lewis *et al* explain this tendency by examining the different positions newspapers assume in the media field, to sustain their cultural and economic capital, and the support of their audiences.^23^ In the current study, this translated into the right-leaning press echoing the sentiments of the government and arguing in favour of in-person care, whereas left-leaning outlets (echoing the comments of many GPs quoted) advocated retaining remote care to some capacity to avoid further strain on an already overstretched workforce. This polarised media landscape shaped public views in 2021, in sharp contrast to a relatively uniform media response on remote consulting in 2020.^8^

Although media views about the potential and limitations of remote consulting shifted as the pandemic progressed, the enduring appeal of attributing agency (positive or negative) to technology remained. In previous work, the authors found that the initial shift to remote care at the start of the pandemic in 2020 was generally supported by the media, which equated technological change with long-overdue progress in service provision.^8^ In 2021, this progress was no longer seen favourably, and remote care was instead blamed for patient harm and inefficiency. This echoes broader technology-focused narratives in COVID-19 with multiple different technologies being presented as ‘solutions’ to pandemic-related challenges, only to scale down expectations later on.^24^

### Implications for research and practice

GP consulting has been gaining momentum as a topic of debate in the media, alongside (mis)representations of GP work and professional identities. But the complex matter of deciding on consultation format has been discussed without the necessary nuances, and without reflecting the complexity of care in general practice.

Negative media coverage matters not just because it is inaccurate and unfair. It may also reduce patients’ confidence in general practice and prevent or delay them seeking care. There is emerging evidence that it also contributes to workforce stress and the retention crisis. A qualitative study of 40 UK GPs conducted in 2020–2021,^25^ and a social media analysis of GPs’ postings during the pandemic by the same authors,^26^ both found that negative media portrayals of general practice (especially the implication that practices were closed when the reality consisted of long hours and high workload) were a significant factor contributing to low morale. The current authors’ own ongoing empirical research has begun to identify similar findings among GPs and their support staff.^27^

More research is needed to understand how different consultation formats, and combinations, can maximise value for different patient groups and different providers. There is significant scope for researchers and practitioners to contribute to media reporting and to broader public engagement around the complexity of remote consulting in general practice. This might help to minimise polarisation, so practitioners and patients can be better supported with situated judgements on consultation formats, as we transition into the next phases of the pandemic.
